# Data from the GIPEyOP online election poll for the 2015 Spanish General election.

**DOI:** 10.1016/j.dib.2020.105719

**Published:** 2020-05-18

**Authors:** José M. Pavía, Vicente Coll-Serrano, Rubén Cuñat-Giménez, Salvador Carrasco-Arroyo

**Affiliations:** a GIPEyOP, UMMICS, Department of Applied Economics, University of Valencia, Spain; b Department of Applied Economics, University of Valencia, Spain

**Keywords:** Spanish General elections, Voting, Public opinion, Election polls, Online surveys

## Abstract

The general elections of 2015 in Spain took place in the middle of the Great Recession after several years of austerity economic policies. This election caused a political earthquake that shook the Spanish party system. During the campaign of that election, GIPEyOP (Elections and Public Opinion Research Group from University of Valencia) conducted a survey to collect relevant data about the electorate beliefs, intentions and motivations. This article describes the data set attained, which comprises 71 variables after removing, to ensure full anonymity, those variables that would potentially allow respondents to be identified. Respondents answered a self-administered online questionnaire and were recruited using chain sampling. A total of 14,261 valid observations were collected between 27^th^ November and 18^th^ December 2015. GIPEyOP employed the data collected up to 14^th^ December to deliver a prediction of the election outcomes during that election campaign. Among other issues, this data set may be reused to assess theories of expectations’ formation, to spot how social networks spread geographically and to measure gender, age and education technological gap of the Spanish population.

Specifications table

**Table:** 

Subject	Social Sciences, Sociology, Political Science
Specific subject area	Social Sciences (general), Public opinion, Political Science
Type of data	csv file
How data were acquired	Data was obtained through a self-administered online questionnaire. LimeSurvey was used to conduct the survey. The questionnaire used to be implemented in the online version is provided as supplementary material with the article (in word format).
Data format	Raw
Parameters for data collection	A snowball or chain sampling method was used to recruit respondents.
Description of data collection	The survey was carried out on occasion of the 2015 Spanish General Election. The survey data were collected over twenty days (between 27^th^ November to 18^th^ December 2015).
Data source location	Country: Spain
Data accessibility	Data file (comma-separated values format, csv file) is supplied as supplementary material with this article.

## Value of the data

• This dataset comprises the second public available largest sample of the 2015 Spanish General Election.
• Social scientists, including sociologists, political scientists and public opinion researchers, may benefit from these data.
• Theories of expectations’ formation and of diffusion of social events can be tested using this dataset.
• Although the dataset contains many standard public opinion variables, this dataset with 71 variables is unique providing non-standard variables; among them, respondents’ beliefs and preferences and dates and times of responses.
• This dataset is an example that valuable information can be extracted from non-random samples.
• Gender, age and education technological gap of the Spanish population may be also studied using these data.

## 1 Data Description

Data was obtained through a self-administered online questionnaire, which was implemented by using LimeSurvey (an open source survey tool). The questionnaire is provided with the article as a supplementary material. Table 1 shows a description of the variables available in the dataset.

**Table 1:** Variables description.

Section	Variable	Description	Values
I	PROV	Province in which the respondent has the right to vote in the election	See Table 2
II	ASSESS.SPAIN	Assessment of the general situation (economic, political, social, etc.) in Spain	0 (very bad) to 10 (very good)
II	MOST.VOTED	Belief in which party will win the election	See Table 3
II	RIVERA	Ciudadanos Party leader's assessment	0 (very bad) to 10 (very good)
II	HERZOG	UPyD Party leader's assessment	0 (very bad) to 10 (very good)
II	SANCHEZ	PSOE Party leader's assessment	0 (very bad) to 10 (very good)
II	IGLESIAS	Podemos Party leader's assessment	0 (very bad) to 10 (very good)
II	RAJOY	PP Party leader's assessment	0 (very bad) to 10 (very good)
II	GARZON	IU Party leader's assessment	0 (very bad) to 10 (very good)
II	PROB.VOTE	Are you going to vote in the election?	1. Yes, for sure. 2. I'll probably vote. 3. Probably not. 4. No, for sure. 5. I haven't decided yet.
III	VOTE.GEN	If the General Election were held tomorrow, which political party do you think you would be most likely to vote for?	See Table 3
III	VOTE.GEN.2	When in doubt, what would be your second choice?	See Table 3
IV	from PORC.J1 to PORC.J15, and PORC.J99	In your opinion, what will be the most likely distribution of votes (as a percentage) in your province in the next general election?	Values between 0 and 100. The sum of the percentages of votes for all political parties (see Table 3) must equal 100. When the sum is 100, the value -999.99 appears in the remaining options. Non-responses are NAs.
V	IDEOLOGY	In politics, the expressions "left" and "right" are often used to identify ideologies. Ideologically, where would you stand?	0 (extreme left) to 10 (extreme right)
V	IDEO.PARTY.J1 to IDEO.PARTY.J15, and IDEO.PARTY.J99	Ideological location of political parties (see Table 3)	0 (extreme left) to 10 (extreme right)
VI	BEHAVE.EUR	Did you vote in the 2014 European elections?	1. I didn't vote because I wasn't old enough to vote. 2. I couldn't vote. 3. I usually prefer not to vote. 4. I usually don't vote in European elections. 5. I voted.
VI	EUR2014	Which party did you vote for in the 2014 European elections?	See Table 4
VI	BEHAVE.GEN	Did you vote in the 2011 General election in Spain?	1. I went to vote and I voted. 2. I wasn't old enough to vote. 3. I went to vote, but I didn't vote. 4. I didn't vote, because I couldn't do it. 5. I didn't have the right to vote. 6. I decided not to vote. 7. I don't remember.
VI	GEN2011	Which party did you vote for in the 2011 General election?	See Table 4
VI	BEHAVE.AUT	Did you vote in the last Regional elections?	1. I went to vote and I voted. 2. I wasn't old enough to vote. 3. I went to vote, but I didn't vote. 4. I didn't vote, because I couldn't do it. 5. I didn't have the right to vote. 6. I decided not to vote.
VI	AUT	Which party did you vote for in the last Regional elections? (Note: 2012 or 2015 depending on the Region, the Autonomous Community)	See Table 4
VII	POSTAL.CODE	Postal code	Full digits postal code
VII	YEAR	Year of birthday	Number between 1900 and 2015
VII	GENDER	Gender of the respondent	1. Male. 2. Female.
VII	EDUCATION	Highest education level achieved	1. No formal education. 2. Primary education. 3. Secondary education. 4. Certificate of Higher Education (HNC). 5. University Degree.
VII	ACTIVITY	Employment situation of the respondent	1. Working (employed or self-employed). 2. Retired (previously worked). 3. Retired (not previously employed). 4. Unemployed and previously employed. 5. Looking for your first job. 6. Student. 7. Unpaid domestic work. 8. Another situation.
VII	INCOMES	Monthly income (including all members in the household)	1. Without incomes. 2. Less than 300€. 3. From 301 to 600€. 4. From 601 to 900€. 5. From 901 to 1200€. 6. From 1201 to 1800€. 7. From 1801 to 2400€. 8. From 2401 to 3000€. 9. From 3001 to 4500€. 10. From 4501 to 6000€. 11. More than 6000€.
VIII	DEVICE	Electronic device used to answer the questionnaire	1. Desktop computer. 2. Laptop. 3. Tablet. 4. Mobile phone. 5. Other.
VIII	DISSEMINATION	Means of dissemination of the survey	1. Email. 2. WhatsApp. 3. Media system. 4. Facebook. 5. Twitter. 6. LinkedIn. 7. Other.
VIII	ACCESS	Means by which the questionnaire has reached the respondent	1. It was sent to me by an acquaintance. 2. I have accessed it through references from the University of Valencia. 3. It was sent to me by someone I don't know. 4. I have accessed it through references in the media. 5. I have accessed it through references in the media. 6. Other.
	START.TIME	When the questionnaire was started	Date and time
	END.TIME	When the questionnaire was finished	Date and time
	DURATION	Time taken to complete the questionnaire	Number of seconds taken.
	TIME.I	Time needed to complete section I	Number of seconds taken.
	TIME.II	Time needed to complete section II	Number of seconds taken.
	TIME.III	Time needed to complete section III	Number of seconds taken.
	TIME.IV	Time needed to complete section IV	Number of seconds taken.
	TIME.V	Time needed to complete section V	Number of seconds taken.
	TIME.VI	Time needed to complete section VI	Number of seconds taken.
	TIME.VII	Time needed to complete section VII	Number of seconds taken.
	TIME.VIII	Time needed to complete section VIII	Number of seconds taken.

As we can see in Table 1, the values of the variable PROV (section 1) correspond to the Spanish provinces (see Table 2). In the questionnaire, the respondent had to select the province in where she/he had the right to vote, not her/his province of residence.

**Table 2:** Provinces of Spain.

Province	Province
ALBACETE	LEÓN
ALICANTE/ALACANT	LLEIDA
ALMERÍA	LUGO
ARABA/ÁLAVA	MADRID
ASTURIAS	MÁLAGA
ÁVILA	MURCIA
BADAJOZ	NAVARRA
BALEARS, ILLES	OURENSE
BARCELONA	PALENCIA
BIZKAIA	PALMAS, LAS
BURGOS	PONTEVEDRA
CÁCERES	RIOJA, LA
CÁDIZ	SALAMANCA
CANTABRIA	SANTA CRUZ DE TENERIFE
CASTELLÓN/CASTELLÓ	SEGOVIA
CIUDAD REAL	SEVILLA
CÓRDOBA	SORIA
CORUÑA, A	TARRAGONA
CUENCA	TERUEL
GIPUZKOA	TOLEDO
GIRONA	VALENCIA/VALÈNCIA
GRANADA	VALLADOLID
GUADALAJARA	ZAMORA
HUELVA	ZARAGOZA
HUESCA	CEUTA
JAÉN	MELILLA

Section III of the questionnaire asked two questions: (i) If the General Elections were held tomorrow, which political party or electoral alliance would your vote for? (variable VOTE.GEN), and (ii) When in doubt, what would be your second choice? (variable VOTE.GEN.2). These questions were conditional questions since not all political parties were running in all provinces. Depending on the province in where the respondent had the right to vote, different political parties were shown as an answer option to the respondent. Table 3 shows the main political parties running in the 2015 Spanish General election with the identification code included in the dataset.

**Table 3:** Codes of political parties in 2015 General Election.

Code	Political party
J1	PP
J2	PSOE
J3	CIUDADANOS
J4	PODEMOS
J5	UP: IU-UPeC
J6	UPyD
J7	ERC-CATSÍ
J8	EAJ-PNV
J9	UNIÓ.CAT
J10	PACMA
J11	DL (CONVERGÈNCIA)
J12	EH-Bildu
J13	NÓS
J14	GBAI
J15	CCa-PNC
J99	Other options

Similarly, section VI asked three questions (see the questionnaire) about the political party that the respondent voted for in the 2014 European elections (variable EUR2014), in the 2011 General election (variable GEN2011), and in the last Regional elections (variable AUT). Table 4 shows the main political parties that were running in these elections with their corresponding identification code in the dataset.

**Table 4:** Codes of political parties in several elections.

2014 European elections		2011 General election		2015 Regional Elections	
Code	Political party	Code	Political party	Code	Political party
E1	PP	G1	PP	A1	PP
E2	PSOE	G2	PSOE	A2	PSOE
E3	IU	G3	IU	A3	PODEMOS
E4	UPyD	G4	UPyD	A4	C's
E5	PODEMOS	G5	COMPROMÍS-Q	A5	IU
E6	CIUDADANOS	G6	EQUO	A6	COMPROMÍS
E7	PRIMAVERA EUROPEA	G7	AMAIUR	A8	EH BILDU
E8	EH Bildu	G8	EAJ-PNV	A9	UPYD
E9	EAJ-PNV	G9	FAC (FORO)	A10	FAC (FORO)
E10	FAC	G10	ERC-RI.cat	A11	MÉS
E11	VOX	G11	PxC	A12	EL PI
E12	EPDD	G12	CiU	A13	MpM
E13	ERC-NECat-EPDD	G13	PA	A14	EAJ-PNV
E14	CiU	G14	PRC	A15	UPN
E15	PARTIDO ANDALUCISTA	G15	BNG	A16	EX
E16	AGE	G16	GBAI	A17	PA
E17	BNG	G17	CC-NC-PNC	A18	P.R.C.
E18	CCa-PNC	G18	CABALLAS	A19	BNG
E19	PACMA	G19	Other options	A20	PAR
E20	EB			A21	CHA
E21	Other options			A22	UPL
				A23	IP
				A24	Geroa Bai
				A25	CCa-PNC
				A26	PR+
				A27	CI-CCD
				A28	AHORA DECIDE/AS
				A29	ADEIZA
				A30	Caballas
				A31	MDyC
				A32	CpM
				A33	PPL
				A34	Otra opción
				A35	JxSí
				A36	CatSiqueesPot
				A37	Unió
				A38	CUP
				A39	NCa
				A40	UNIDOS
				A99	Other options

Data was collected between 27^th^ November and 18^th^ December 2015. The dataset, which is provided with the article, contains a total of 14,261 valid observations of 71 variables (see Table 1). Table 5 shows the distribution of the sample sizes by province and Table 6 the distribution by Autonomous Community.

**Table 5:** Sample size by province.

Province	Sample size	Province	Sample size
ALBACETE	152	JAEN	69
ALICANTE	732	LEON	52
ALMERIA	85	LLEIDA	37
ALAVA	33	LUGO	44
ASTURIAS	204	MADRID	1625
AVILA	21	MALAGA	136
BADAJOZ	66	MELILLA	21
BALEARS, ILLES	152	MURCIA	275
BARCELONA	615	NAVARRA	123
BIZKAIA	124	OURENSE	30
BURGOS	49	PALENCIA	14
CACERES	54	PALMAS, LAS	85
CADIZ	150	PONTEVEDRA	111
CANTABRIA	96	RIOJA, LA	175
CASTELLON	456	SALAMANCA	110
CEUTA	6	SANTA CRUZ DE TENERIFE	100
CIUDAD REAL	80	SEGOVIA	14
CORDOBA	99	SEVILLA	225
CORUNA, A	224	SORIA	25
CUENCA	87	TARRAGONA	58
GIPUZKOA	95	TERUEL	70
GIRONA	45	TOLEDO	187
GRANADA	128	VALENCIA	6475
GUADALAJARA	49	VALLADOLID	92
HUELVA	35	ZAMORA	21
HUESCA	45	ZARAGOZA	205

**Table 6:** Sample size by Autonomous Community.

Region	Sample size	Region	Sample size
España	14261	Comunidad de Madrid	1625
Andalucía	927	C. Foral de Navarra	123
Aragón	320	Comunitat Valenciana	7663
Canarias	185	Extremadura	120
Cantabria	96	Galicia	409
Castilla-La Mancha	555	Illes Balears	152
Castilla y León	398	La Rioja	175
Cataluña	755	País Vasco	252
Ciudad de Ceuta	6	Principado de Asturias	204
Ciudad de Melilla	21	Región de Murcia	275

## 2 Experimental Design, Materials, and Methods

The Internet has been a real revolution that is opening up very interesting research possibilities for social scientists. Thus, it is not surprising that we are witnessing the emergence of new experiences, mainly from the academic world, which, exploiting the possibilities of the Internet, seek to demonstrate that it is also possible to generate quality predictions with biased samples. From the use of responses collected from Xbox users [1] to employing mechanisms where the potential respondent population is not selected by the pollster, but rather the respondents self-select. Thus, during the campaign for the 2015 General Election in Spain on 20^th^ December, the research group GIPEyOP (http://gipeyop.uv.es/) carried out an experience of this nature: a self-administered online questionnaire was released and a snowball (or chain-referral) sampling was used [2].

We launched the questionnaire from Valencia via email and social networks such as WhatsApp, Facebook, Twitter, etc. In our message we asked for the collaboration of the respondents so that they could distribute, at the same time, the questionnaire among their acquaintances, friends and family. Each of the questionnaires received was subjected to an intense filtering process to select only those questionnaires with a minimum quality (internal consistency) and quantity requirements in the available information. Among other issues, (i) we controlled that the responses were made from a Spanish IP address, and (ii) we compared the responses collected with two electronic versions of the questionnaire where we set different specifications about the number of attempts available and we assessed the consistency of respondents considering variables like leaders’ assessment, ideology or vote intention. These actions lead us to discard 4,544 responses. The validated dataset contains a total of 14,261 observations of 71 variables (see Table 1).

### 2.1 Data Quality

The data available cannot be considered as a simple random sample and it is difficult to consider it as a representative sample. The collection method means that the selection procedure necessarily introduces coverage and self-selection bias into the sample. The question of the theoretical non-representativeness of the sample does not constitute a differential fact of our data. All electoral opinion samples suffer to a greater or lesser extent from the problem of representativeness, mainly due to the differential non-response rates that pollsters encounter during fieldwork [3]. This problem even happens to the more respected pollsters, such as the Centro de Investigaciones Sociológicas (CIS), the most prestigious Spanish survey organization [4]. As a random selected example, we can consider the barometer conducted by CIS in October 2014, when comparing collected raw answers and related actual data, we observe that just 28% of the respondents claimed to have voted for Popular Party (PP) in the 2011 Spanish General Election [5], when actually 45% of voters supported PP in that election. Similarly, the raw data available in our dataset has different sources of bias, as it can be observed in Table 7.

**Table 7:** Actual and Dataset distributions for some regional and national level available registers.

Territorial Distribution			Demographic Distribution			Political Distribution		
Region	Population	Dataset	Age Groups	Population	Dataset	Election option	Official Results	Dataset
Andalucia	18.14%	6.50%	18_25	9.30%	26.39%	PSOE	18.68%	20.05%
Aragon	2.85%	2.24%	26_30	6.43%	10.99%	PP	28.98%	10.78%
Canarias	4.43%	1.30%	31_35	7.89%	10.13%	IU	4.50%	19.72%
Cantabria	1.35%	0.67%	36_40	9.80%	10.02%	UPyD	3.05%	4.69%
Castilla-La Mancha	4.45%	3.89%	41_45	9.93%	8.58%	CiU	2.71%	0.52%
Castilla y Leon	5.77%	2.79%	46_50	9.65%	9.00%	EAJ-PNV	0.87%	0.27%
Catalunya	15.38%	5.29%	51_55	9.12%	8.19%	AMAIUR	0.89%	0.46%
Ciudad de Ceuta	0.17%	0.04%	56_60	8.06%	6.93%	BNG	0.49%	0.58%
Ciudad de Melilla	0.15%	0.15%	61_65	6.83%	5.04%	GBAI	0.11%	0.04%
Comunidad de Madrid	13.40%	11.39%	66_70	6.43%	3.08%	ERC-RI.CAT	0.69%	0.87%
Comunidad Foral de Navarra	1.38%	0.86%	71_75	5.30%	1.23%	PA	0.21%	0.05%
Comunitat Valenciana	10.18%	53.73%	over_75	11.26%	0.42%	CC-NC-PNC	0.38%	0.03%
Extremadura	2.55%	0.84%				COMPROMÍS-Q	0.33%	10.28%
Galicia	6.55%	2.87%				FAC (FORO)	0.27%	0.11%
Illes Balears	2.16%	1.07%	**Gender**	**Population**	**Dataset**	PRC	0.12%	0.02%
La Rioja	0.68%	1.23%	Men	48.34%	64.76%	Others	3.52%	8.42%
Pais Vasco	4.97%	1.77%	Women	51.66%	35.24%	Abstention	29.64%	9.02%
Principado de Asturias	2.53%	1.43%				New electors	4.57%	14.10%
Region de Murcia	2.90%	1.93%						

Demographic Population data come from INE (www.ine.es).

Election results come from GIPEyOP (gipeyop.uv.es/).

2011 General Election results have been adjusted to add 100% after taking into account new electors in 2015.

Others include blank and null votes.

In Table 7 we compare, for some variables, sample data aggregations with actual register data and, as it is obvious, different subgroups of population were overrepresented (like the people living in the Valencian region), whereas other groups were underrepresented (such as the PP voters). This does not mean that not valuable information can be derived from the data available. As an example, during the election campaign, on 14^th^ December 2015, the last day to release polls to the public according to the Spanish electoral law, GIPEyOP delivered a prediction for the election outcomes and the estimates made by GIPEyOP were among the top-ten most accurate predictions published during that electoral campaign. In particular, it was the sixth out of 28 poll-based published vote estimates of the 2015 General Election.

GIPEyOP estimates were built after amending the major deviations presented in the collected data by constructing vote propensities using socio-demographic variables and reported recall votes. Particularly, the prediction methodology of the GIPEyOP survey was based on the estimation (through the use of multilevel models) of the probabilities that each person has of voting for each party based on her/his individual variables and the characteristics of the environment where she/he lived. As individual characteristics, the following variables (see Table 1) available from the questionnaire were considered: age, sex, level of studies and voting history of the surveyed person; while, as regards contextual characteristics, the model included the province of residence, the demographic structure of the province (as regards the distribution of the population by municipality size and by age groups) and the Autonomous Community.

The example above shows that, by properly weighting the responses, the dataset described in this paper can be used to make accurate population inferences. For example, the interested reader may use the marginal distributions in Table 7 not only to assess the level of bias in our dataset, but also to calibrate the sample and, what's more, she/he may employ the accompanied Appendix file (Excel file supplied as supplementary material) to construct weights from the joint distributions. Likewise, in our view, when constructing individual level models, the biases presented in the dataset could be overcame just by working conditionally, i.e., by including the biased features as explanatory variables in the model. This dataset therefore could be reused to assess theories of expectations’ formation [6], to spot how social networks spread geographically or to measure gender, age and education technological gaps of the Spanish population.

## Declaration of Competing Interest

The authors declare that they have no known competing financial interests or personal relationships which have, or could be perceived to have, influenced the work reported in this article.

## Appendix Supplementary materials

Image, application 1

Image, application 2

Image, application 3

Image, application 4
