# Supplementary material for: Data from the GIPEyOP online election poll for the 2015 Spanish General election
Source: Data Brief. 2020 May 18;31:105719. doi: 10.1016/j.dib.2020.105719 (PMC7256455; doi:10.1016/j.dib.2020.105719)
Supplement: Supplementary file 1 [file mmc1.docx]

**2015 GENERAL ELECTIONS**

**QUESTIONNAIRE**

**Section I**

**1. Please, select the province in where you have the right to vote in the next General Elections. (This question is mandatory to access the rest of the questionnaire)**

**Section II**

**2. To begin with, how would you rate the overall situation in Spain? (from 0 = very bad) to 10 = very good) [Please click on the square and move the cursor to the desired score]**

**3. As you know, on 20 December the General Elections will be held. Which party do you think will be the most voted in Spain?**

- PP
- PSOE
- CIUDADANOS
- PODEMOS
- UNIDAD POPULAR
- UPyD
- Another option

**4. Could you rate the following candidates (from 0 = very bad to 10 = very good)? If you do not know any candidate, you do not need to rate him/her. [Please click on the square and move the cursor to the desired score]**

Mariano Rajoy

Pedro Sánchez

Pablo Iglesias

Albert Rivera

Alberto Garzón

Andrés Herzog

**5. Do you have the intention to vote? (Please select only one of the following options)**

- Yes, for sure
- I will probably vote
- Probably not
- No, for sure
- I have not decided yet

**Section III**

**6. If the General Elections were held tomorrow, which party or electoral alliance would you vote for? (Conditional question by province)**

- First choice
- If you are in doubt, what would be your second choice?

**Section IV**

**REMEMBER: The sum of the percentages of votes for all parties must be equal to 100.**

**7. In your opinion, what will be the most likely (percentage) distribution of votes in your province in the next elections? (Conditional question by province)**

**Section V**

**8.  In politics, the expressions "left" and "right" are often used. Ideologically, where would you place yourself? [Being 0 extreme left and 10 extreme right]**

**9. Where would you place the following parties? [from 0 = extreme left to 10 = extreme right] (Conditional question by province)**

**9A. On the axis of national identity, from “I feel myself only Catalonian” to “I feel myself only Spanish”, could you indicate where you would place yourself? (Conditional question for only Catalonian provinces)
Catalan only/ Spanish only (slide bar)**

**9B. On the axis of national identity, from “I feel myself only Basque” to “I feel myself only Spanish”, could you indicate where you would place yourself? (Conditional question for only Basque country provinces)
Basque only/Spanish only (slide bar)**

**9C On the axis of national identity, from “I feel myself only Galician” to “I feel myself only Spanish”, could you indicate where you would place yourself? (Conditional question for only Galician provinces)
Galician only/Spanish only (slide bar)**

**Section VI**

**Now, we are going to ask you some questions aimed at trying to correct any biases that may occur as a result of both the survey mode (self-administered online survey) and the selection process used (snowball).**

**10. Many people have told us that they did not want or could not vote in the past European elections. If so, which of the following statements best suits your situation? Please, select only one of the following options:**

- I didn't vote because I wasn't old enough to vote.
- I couldn´t vote.
- I usually prefer not to vote.
- I do not usually vote in the European elections.
- I voted.

**10A. Could you select the party or coalition you voted for in the past European Elections in 2014? (Conditional question by province)**

**11.** **And what did you do in the past General Elections held in 2011? Please select only one of the following options:**

- I went to vote and I voted.
- I wasn't old enough to vote.
- I went to vote, but I didn’t vote.
- I didn't vote, because I couldn't do it.
- I didn't have the right to vote.
- I decided not to vote
- I don`t remember.

**11A. Could you select the party or electoral alliance you voted for in the past General Elections in 2011? (Condition by provinces)**

**12. Could you point out what you did in the last Autonomous Elections held in 2015? Please select only one of the following options:**

- I went to vote and I voted.
- I wasn't old enough to vote.
- I went to vote, but I didn’t vote.
- I didn't vote, because I couldn't do it.
- I didn't have the right to vote.
- I decided not to vote.

**12A. Could you select the party or electoral alliance you voted for in the past Autonomous Elections in 2015? (Conditional question by province)**

**Section VII**

**13. Could you enter your postal code?**

**14. Could you enter your year of birth? Your answer must be between 1900 and 2015.**

**15. Gender**

- Male
- Female

**16. Highest education level achieved. Please select only one of the following options:**

- No formal education.
- Primary education.
- Secondary education.
- Certificate of Higher Education (HNC).
- University Degree.

**17. What is your current employment situation? Please select only one of the following options:**

- Working (employed or self-employed).
- Retired (worked previously).
- Retired (did not work previously).
- Unemployed and was previously employed/and worked previously
- Unemployed and looking for your first job.
- Student.
- Unpaid domestic work.
- Another situation.

**18. Please, could you place the monthly income band in your household? (including all members). Please select only one of the following options:**

- You have no income of any kind.
- Less or equal to 300 €
- From 301 to 600 €
- From 301 to 600 €
- From 601 to 900 €
- From 901 to 1.200 €
- From 1.201 to 1.800 €
- From 1.801 to 2.400 €
- From 2.401 to 3.000 €
- From 3.001 to 4.500 €
- From 4.501 to 6.000 €
- More than 6.000 €

**Section VIII**

**19. Electronic device used to answer the survey:**

**Please select only one of the following options:**

- Desktop computer.
- Laptop.
- Tablet.
- Mobile phone.
- Other.

**20. Could you select the means by which you received the questionnaire?**

**Please select only one of the following options:**

- Email.
- WhatsApp.
- Mass media
- Facebook.
- Twitter.
- LinkedIn.
- Other.

**21. Means of access. Could you please tell us how you got this questionnaire?
Please select only one of the following options:**

- It was sent to me by an acquaintance.
- I have accessed it through references from the University of Valencia.
- It was sent to me by a someone I don't know.
- I have accessed it through references in the media.
- I have accessed it through references in the media.
- Other.
